# Separation of Nano- and Microparticle Flows Using Thermophoresis in Branched Microfluidic Channels

**DOI:** 10.3390/mi10050321

**Published:** 2019-05-12

**Authors:** Tetsuro Tsuji, Yuki Matsumoto, Ryo Kugimiya, Kentaro Doi, Satoyuki Kawano

**Affiliations:** Graduate School of Engineering Science, Osaka University, Toyonaka, Osaka 560-8531, Japan; tsuji@me.es.osaka-u.ac.jp (T.T.); ymatsumoto@bnf.me.es.osaka-u.ac.jp (Y.M.); zeath.00@gmail.com (R.K.); doi@me.es.osaka-u.ac.jp (K.D.)

**Keywords:** microscale thermophoresis, multiphase flow, microfluidic channels, nano/microparticle separation, micro-electro-mechanical-systems (MEMS) technologies

## Abstract

Particle flow separation is a useful technique in lab-on-a-chip applications to selectively transport dispersed phases to a desired branch in microfluidic devices. The present study aims to demonstrate both nano- and microparticle flow separation using microscale thermophoresis at a Y-shaped branch in microfluidic channels. Microscale thermophoresis is the transport of tiny particles induced by a temperature gradient in fluids where the temperature variation is localized in the region of micrometer order. Localized temperature increases near the branch are achieved using the Joule heat from a thin-film micro electrode embedded in the bottom wall of the microfluidic channel. The inlet flow of the particle dispersion is divided into two outlet flows which are controlled to possess the same flow rates at the symmetric branches. The particle flow into one of the outlets is blocked by microscale thermophoresis since the particles are repelled from the hot region in the experimental conditions used here. As a result, only the solvent at one of outlets and the residual particle dispersion at the other outlet are obtained, i.e., the separation of particles flows is achieved. A simple model to explain the dynamic behavior of the nanoparticle distribution near the electrode is proposed, and a qualitative agreement with the experimental results is obtained. The proposed method can be easily combined with standard microfluidic devices and is expected to facilitate the development of novel particle separation and filtration technologies.

## 1. Introduction

Fluids that contain dispersed phases, such as nano- and microparticles, appear in a wide range of applications. For instance, a nanofluid is a class of fluid that contains nanometer-sized materials, in which a significant increase in the heat-transfer rate compared to conventional engineered fluid has been reported [1,2]. Ever since its discovery, the nanofluid has served in several engineering applications, e.g., fuel-cells [3,4,5], porous materials [6,7,8], and petroleum engineering applications [9,10,11,12]. In addition to these applications, nanofluids have become an important research topic in the development of lab-on-a-chip (LOC) devices, where the sorting and/or accumulation of target nanomaterials in fluidic devices are necessary for a controlled chemical reaction or an analysis of the targets [13,14]. In LOC devices, controlling and separating the flow of tiny dispersed phases, such as nanoparticles in nanofluids, is one of the main concerns in the field of microfluidics and nanofluidics [15,16,17,18,19,20,21]. To be more specific, flow control of nanoparticles can enhance the detection/identification performance of the biosensor [22] installed in a LOC device. Therefore, various techniques to control the particle flow in microfluidic channels have been proposed in [15,16,17,18,19,20]. For instance, electrophoresis [23,24] and dielectrophoresis [25] in micro- and nanofluidic devices are widely acknowledged experimental techniques using electrokinetic effects. However, applying electrical potential differences across the device may result in electroosmotic flows and/or the electrolysis of solvents, which may complicate data analysis and be undesirable depending on a situation. Other than these electrokinetic methods, other driving mechanisms such as diffusiophoresis [26,27,28,29,30] and thermophoresis [31,32,33] have also been actively investigated recently. Since each method has a different physical basis, one may choose a suitable technique according to the properties of the target materials.

Among these driving mechanisms, thermophoresis is expected to introduce a new direction for particle flow control since it is sensitive to the composition of a continuous phase and the nature of particle characteristics, such as chemical surface modifications. More specifically, the direction of particle motion may be controlled by choosing a proper experimental setting. For instance, it has been shown that the direction of thermophoresis can be reversed by controlling the average temperature of the solution [34,35,36] or by adding electrolytes [37,38,39,40] or polymers [41,42] into the solution. Owing to the high sensitivity of thermophoresis, depending on the nature of particle and/or solvent, microscale thermophoresis has recently been developed to evaluate protein-binding [43,44]. Here, the prefix terminology “microscale” is used to emphasize that the driving temperature distribution is localized in a microscale spatial region. However, although there have been theoretical [33,45,46,47,48] and simulation-based [49,50,51,52] approaches to understand the nature of thermophoresis, its physical mechanism is not yet fully understood.

Given such a growing interest in thermophoretic manipulation, the previous study by the authors tried to apply microscale thermophoresis to particle flow control in LOC devices [53,54]. More specifically, using micro-electro-mechanical-systems (MEMS) technologies, a micro heater was installed in a straight microfluidic channel, and the on-chip thermophoretic separation device was developed [53]. The counterbalance between the flow of the continuous phase and the thermophoresis of microparticles resulted in the formation of a localized particle distribution in the straight channel. However, the particle flow separation to branched channels, as demonstrated in [15,16,17,19,20], was not achieved in the previous study. It is useful in LOC applications to transport dispersed phases to a desired branch. The present paper is an extension of the previous study [53] on branched microfluidic channels, which are more suitable for the separation of a dispersed phase from a continuous one. To eliminate unnecessary complexity, a symmetric Y-shaped branch is used as a microfluidic channel. In this way, the effect of thermophoresis on the particle flow separation is elucidated. Moreover, a simple numerical model is introduced to explain the separation dynamics at the Y-shaped branch. In the present study, the size of the particle is reduced from microscale to nanoscale to demonstrate that the present approach is also applicable not only to cells with *O*(1) μm but also to viruses [55,56] or pollen allergen particles [57] with O(10)–O(100) nm, broadening the scope of application of state-of-the-art micro- and nanofluidics in biosciences. The present demonstration of nano- and microparticle flow separation using thermophoresis suggests the function of selective particle flow control may feasibly be installed to existing microfluidic devices.

## 2. Experimental Methods

### 2.1. Details of Microfluidic Devices

**Figure 1:**
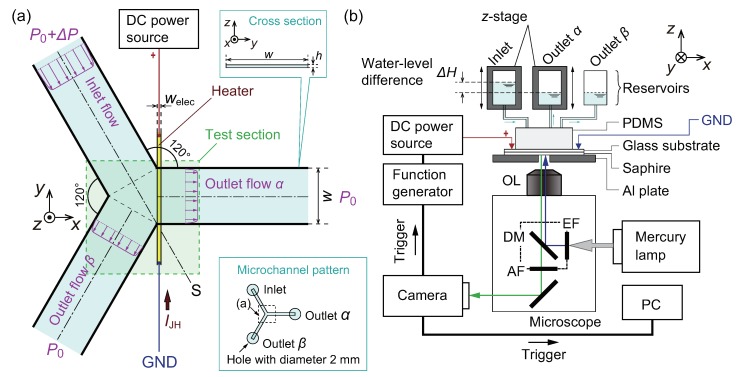
(**a**) Schematic of the test section. The branched microfluidic channel has a rectangular cross-section in the yz plane with a height h=17.2 μm and a width w=450 μm. The inlet flow is divided into two outlet flows α and β. A thin-film electrode heater is fabricated at the entrance of the outlet flow α. Flow profiles of the inlet and outlets are schematically drawn based on the analytical solution of the Poiseuille flow in a rectangular channel [58]. (**b**) Overview of the experimental setup. EF: emission filter. AF: absorption filter. DM: dichroic mirror. OL: objective lens. PC: personal computer.

Microfluidic channels are fabricated by bonding a block made of polydimethylsiloxane (PDMS) and a glass substrate using a similar protocol to that described in the previous study [53]. In the present paper, the PDMS block has a Y-shaped branch in a microfluidic channel. The schematic of the test section near the branch is shown in Figure 1a. The channel has a uniform cross-section with a height h=17.2 μm and a width w=450 μm. The dimension of the cross-section of the channel is similar to that in the previous study [53], where unwanted thermal convection was confirmed to be absent thanks to the small channel height. Three holes with a diameter 2 mm for an inlet and two outlets are fabricated, as shown in the inset of Figure 1a. These holes are connected to reservoirs using silicone tubes, as shown in Figure 1b. The distance from the holes to the Y-shaped branch, i.e., the channel lengths, is L=5 mm for the inlet and two outlets. This technique is considered in terms of the fluid dynamics of the continuous phase to realize a fully developed flow at the test section and almost equal flow rates in two branched outlets. To induce a temperature increase for thermophoresis of the dispersed phase, an electrode heater, which has the boundary condition of uniform heat flux, is used, as shown in Figure 1a, where the electrode width is $w_{\mathrm{elec}}=20$ μm and thickness is 150 nm. The electrode thickness is thin compared to the channel height *h*, and it does not affect the flows. The fabrication process is described in the following.

#### 2.1.1. Fabrication of a PDMS Block

A mold for the microfluidic channel pattern is prepared on a Si substrate by a photolithography of negative photoresist SU-8 3005 (MicroChem Corp., Westborough, MA, USA). The PDMS block is cast from the mold to obtain the microfluidic channel pattern. The height of the mold, which determines the height of the microfluidic channel *h*, is measured as h=17.2±0.2 μm by scanning the PDMS block using a laser displacement sensor (LK-H008W, Keyence, Osaka, Japan).

#### 2.1.2. Fabrication of the Electrode Pattern on the Glass Substrate

The glass substrate is sonicated in dimethylformamide (DMF), ethanol, and ultra-pure water in series for 15 min each. After drying out the glass substrate at 200 ${}^{\circ }$C for 5 min, an Au thin-film is deposited on the substrate by sputtering, where the thickness of the Au layer is 150 nm. Here, Cr is used as an adhesion layer between the glass and Au. A positive photoresist (AZ5214E, Merck, Germany) is spin-coated onto the substrate. After a prebake at 90 ${}^{\circ }$C for 2 min, the substrate is exposed to UV light with 20 mJ·cm${}^{-2}$ through a photomask to obtain an electrode pattern. The substrate is then immersed in a developer solution (a mixture of 1:1 ultra-pure water and AZ developer; Merck, Germany) to obtain the photoresist layer with the electrode pattern. After the postbake at 120 ${}^{\circ }$C for 2 min, the substrate is immersed into etching solutions for Au (AURUM-301, Kanto Chemical Co., Inc., Tokyo, Japan) and Cr (Mixed-Acid Cr Etching solution, Kanto Chemical Co., Inc., Tokyo, Japan) layers. Finally, the substrate is sonicated in acetone and ultra-pure water.

The electrode heater has an electrical resistance of 23.3±1.7 Ω, i.e., the variation in the fabrication error is 7%. The error may be attributed to the presence of a gap between the photomask and the substrate during UV exposure, which determines the accuracy of the pattern transfer; this is difficult to control precisely using a manual mask aligner such as that which is used in the present fabrication. For the present research, the error is within the acceptable range, but a direct pattern exposure system, such as a laser lithography system, will be necessary to achieve further miniaturization of the electrode.

#### 2.1.3. Bonding Process

The contact surfaces of the PDMS block and the glass substrate are treated by oxygen plasma (RIE-10NR, Samco, Kyoto, Japan) to enhance the adhesion. The bonding process is carried out using an aligner so that the electrode is placed at the entrance of the outlet α, as shown in Figure 1a. A direct current (DC) power source (PAN35-10A, Kikusui Electronics Corp., Yokohama, Japan) is connected to the electrode. An electric current $I_{\mathrm{JH}}$ produces the Joule heat from the electrode and a microscale temperature distribution is formed at the entrance of the outlet α. In the previous study [53], where the dimensions of the electrode were the same as that of the present study, the maximum temperature $T_{\max}$ near the electrode was measured to be about 360 K. Therefore, a similar temperature increase is expected in the present device. Finally, the inlet and outlets are connected to the reservoirs by silicone tubes.

### 2.2. Experimental Setup

The complete experimental setup is shown in Figure 1b. An inverted microscope (IX-71, Olympus, Tokyo, Japan) with an objective lens (OL, 10x magnification, numerical aperture = 0.3) and a scientific complementary metal-oxide-semiconductor (sCMOS) camera (Zyla 5.5, Andor Technology Ltd., Tokyo, Japan) are used for observation of the device. To prevent the overall temperature increase of the device, it is placed on sapphire glass, which has high thermal conductivity and optical transmissivity. The DC power source is controlled by a function generator (WF1973, NF, Kanagawa, Japan). A trigger signal from the camera synchronizes the image acquisition in a personal computer (PC) and the onset of Joule heating through the function generator.

A mercury lamp (U-HGLGPS, Olympus, Tokyo, Japan) is used as the illumination light source. The illumination light goes through an excitation filter (EF) and is converted to the excitation light. Being irradiated by the excitation light, micro- or nanoparticles in the device emit fluorescence, which is monitored by the camera through an absorption filter (AF).

The flow rate within the device is controlled by water-level differences between the reservoirs. First, the *z*-stage, which holds the reservoir for outlet α, is manipulated to eliminate the water-level difference between the reservoirs for outlets α and β. Then, the particle flow becomes symmetric with respect to a plane, S, shown in Figure 1a. Next, the *z*-stage, which holds the reservoir for the inlet, is manipulated to stop the flow in the microfluidic channel, i.e., all the water-levels in three reservoirs are controlled to be the same. Then, the reservoir for the inlet is lifted by ΔH, as shown in Figure 1b, to induce the fluid flow of a sample solution with a required flow rate. As discussed in [54], the generated pressure difference ΔP in Figure 1 is estimated as ΔP=ρgΔH, where ρ is the mass density of the sample solution and g=9.8 m·s${}^{-2}$ is the acceleration of gravity. In this research, an aqueous solution is used and thus $\rho =1.0\times 10^{3}$ kg·m${}^{-3}$. Because the resolution of the *z*-stage is 1 μm, the resolution of ΔP can be estimated as $1\times 10^{-2}$ Pa. The resulting flow fields will be discussed in Section 3.1.

### 2.3. Sample Solutions

Polystyrene (PS) particles are used as a dispersed phase. In the experiments, PS particles with carboxylate surface modifications in a Tris-HCl aqueous buffer (pH =8.0, 321-90061, Nippon Gene Co., Ltd., Tokyo, Japan) are used since this choice was confirmed to yield thermophoresis in the previous experiments [53] when the particle diameter *d* was 0.99±0.022 μm. The concentration of Tris-HCl is 10 mM. To avoid the occurrence of inter-particle interactions, the concentration of particles should be dilute and is set to be less than $4\;\times 10^{-2}$ wt%. These sample solutions were prepared using ultra-pure water. Microparticle (d=0.99±0.022 μm, F8823, Molecular Probes, Eugene, OR, USA) and nanoparticles (d=99±8 nm, F8803, Molecular Probes, Eugene, OR, USA) are tested in the present paper.

### 2.4. Procedures

As described in Section 2.2, the mean flow in the microfluidic channel is induced, where the flow is symmetric with respect to the plane S in Figure 1a. At t=0 s, heating the microfluidic channel is induced by applying the electric current $I_{\mathrm{JH}}=4\times 10^{-2}$ A, and recording the subsequent behaviors of PS particles. The duration of the experiment is set to 300 s. To focus on the effect of temperature increase, it must be ensured that the pressure difference ΔP does not change during the experiments of 300 s. This is confirmed as follows. As will be shown in Section 3.1, the flow speed in the inlet channel is less than 10 μm·s${}^{-1}$. That is, the flow rate, which is obtained by multiplying the flow speed by the cross-sectional area $wh=7.7\times 10^{3}$ μm${}^{2}$, is estimated as $7.7\times 10^{-14}$ m${}^{3}$·s${}^{-1}$. Due to mass conservation, this flow rate must be compensated by the decrease (and increase) of the reservoir water level in the inlet (and outlets). The cross-section of the reservoir is $2.3\times 10^{-4}$ m${}^{2}$, that is, ΔH decreases with the speed $3.3\times 10^{-10}$ m·s${}^{-1}$ to compensate for the mass flow in the microfluidic channel. For the experiment of 300 s, the difference between ΔH(t=0s) and ΔH(t=300s) is estimated as, at most, $1.0\times 10^{-7}$ m, which corresponds to $\Delta P\left(t=0\;s\right)-\Delta P\left(t=300\;s\right)=\rho g\left[\Delta H\left(t=0\;s\right)-\Delta H\left(t=300\;s\right)\right]\approx O\left(10^{-3}\right)$ Pa. Since ΔP(t=0s)=0.5 or 1.0 Pa is used in the present paper, it is considered that the variation of ΔP is negligibly small in the experiments. In other words, the mean flow can be considered to be in a steady state during the experiments.

## 3. Results and Discussion

### 3.1. Flow Fields

Thermophoresis is a rather weak effect, that is, it can be hindered by the fast flow of continuous phase. Therefore, to observe the effect of thermophoresis effectively, we should induce a creeping flow of O(1)–O(10) μm·s${}^{-1}$ in the microfluidic channel [53,54]. However, in general, the creeping flow is difficult to control since a finer pressure control resolution is required. In this section, the reliability of the control on the flow of the continuous phase is investigated.

**Figure 2:**
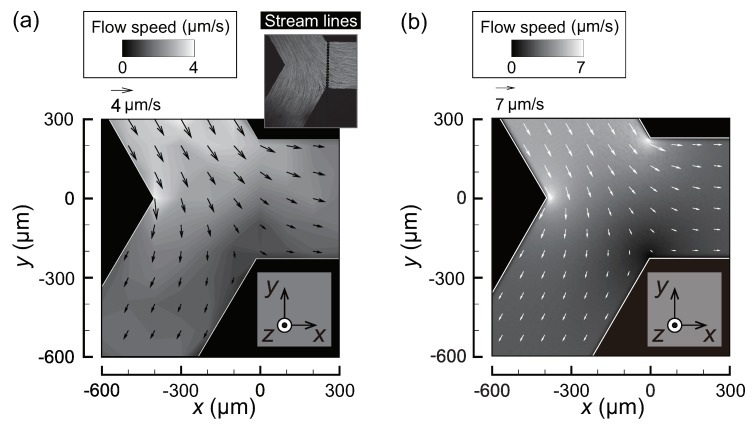
Flow field at the test section without Joule heating for $\Delta P=1\times 10^{-2}$ Pa. The inlet flow is equally separated into two outlet flows. (**a**) Experimental result obtained by the particle image velocimetry (PIV) analysis. (**b**) Numerical result at z=h/2 obtained by the simulation using a finite element method.

The flow field in the absence of temperature increase is presented, where ΔP is set to 1.0 Pa. Figure 2a shows the experimental result obtained by the particle image velocimetry (PIV) using the microparticle as a tracer, where the inset shows corresponding stream lines. Since the channel height h≈17 μm is small, the fluorescence of particles in the entire *z*-direction is recorded by the camera, i.e., the PIV result is considered to be the average in the *z*-direction. The inlet flow is separated at the Y-shaped branch into two symmetric outlet flows. The flow speed is about 4 μm·s${}^{-1}$ and 2 μm·s${}^{-1}$ for the inlet and the outlets, respectively. To validate the experimental results, the flow field obtained from a numerical simulation using a finite element method is shown in Figure 2b. The simulation is carried out by a commercial software, COMSOL Multiphysics 5.3 (COMSOL, Inc., Stockholm, Sweden). The result shows the velocity vector u at a plane z=h/2. The overall flow profile is consistent with the experimental result in Figure 2a, although the magnitude of the flow is larger than that observed in the experiment. For the Poiseuille flow between two parallel plates, the flow speed averaged in the *z*-direction is 2/3-times the maximum at z=h/2. Therefore, multiplying the result in Figure 2b by a factor 2/3 is expected to yield the result in Figure 2a.

Furthermore, the simulation result is validated using the theoretically obtained inlet flow speed $V_{\mathrm{in}}$, which is the flow speed averaged over the cross-section. The channel length is L=5 mm, the experimentally observed flow speed is $V_{\mathrm{in}}=O\left(1\right)-O\left(10\right)$ μm·s${}^{-1}$, and the viscosity of the solution at room temperature is $\eta =8.94\times 10^{-4}$ Pa·s. Then, laminar flow in the microfluidic channel can be assumed, since the Reynolds number is estimated as $Re=\rho V_{\mathrm{in}}L/\eta <6\times 10^{-2}$ and is sufficiently small. Neglecting the minor pressure losses, such as velocity head, entrance loss, and branch loss, the pressure difference ΔP=1 Pa should be compensated by the friction losses along the microfluidic channel. Considering the incompressible and Newtonian fluid with small Reynolds number, the hydraulic resistances of the inlet $R_{\mathrm{in}}$ and that for the outlet $R_{\mathrm{out}}$ are expressed as $R_{\mathrm{in}}=\frac{12\eta L}{h^{3}w}{\left[1-0.630\left(h/w\right)\right]}^{-1}\left(=R_{\mathrm{out}}\right)$, where the Poiseuille flow in a thin square duct is assumed [58,59]. Then, using Bernoulli’s theorem, the relation $\Delta P=R_{\mathrm{in}}Q_{\mathrm{in}}+R_{\mathrm{out}}Q_{\mathrm{out}}$ holds, where $Q_{\mathrm{in}}$ and $Q_{\mathrm{out}}$ are the flow rates in the inlet and outlet, respectively. It should be noted that $Q_{\mathrm{out}}$ values in both outlets are equal due to the symmetric nature of the microfluidic channel. Then, due to the mass conservation, $Q_{\mathrm{in}}=2Q_{\mathrm{out}}$ holds. Therefore, it is concluded that $\Delta P=\left(3/2\right)R_{\mathrm{in}}Q_{\mathrm{in}}$, which turns out to be $Q_{\mathrm{in}}=\left(2/3\right)\Delta P/R_{\mathrm{in}}$. The average flow speed $V_{\mathrm{in}}=Q_{\mathrm{in}}/\left(wh\right)$ given by the above relation is $V_{\mathrm{in}}=3.5$ μm·s${}^{-1}$. This theoretical value overestimates the simulation value 3.14 μm·s${}^{-1}$ by 10%, where the simulation value is evaluated at the position (x,y)= (−320 μm, 280 μm) in the inlet. The overestimation may be due to the neglected minor losses. Nonetheless, the agreement among the experiment, the simulation, and the theory is reasonable, and it is concluded that the control of the continuous phase is adequate to carry out the thermophoresis experiments.

### 3.2. Microparticle Flow Separation

In this section, the result for microparticles with d=1 μm using the pressure difference ΔP = 1.0 Pa is presented. As demonstrated in the previous work [53], the PS microparticles under the present experimental conditions were thermophobic, that is, the particles were repelled from the hot region. Therefore, it was expected that the particles would move away from the thin-film electrode.

**Figure 3:**
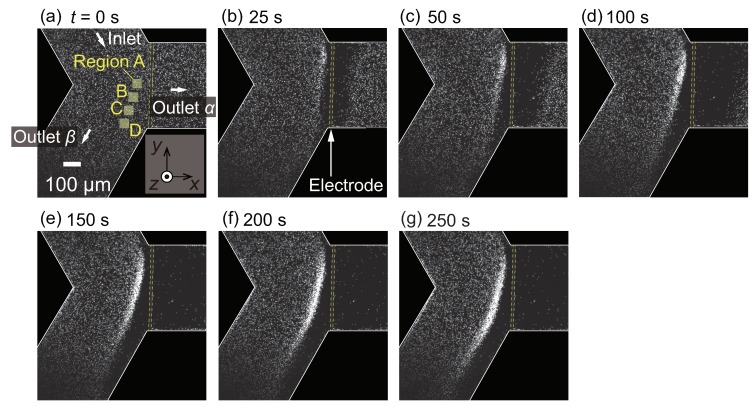
(**a**–**g**) Time series of the particle flow separation induced by microscale thermophoresis for the case with a particle diameter d=1 μm and ΔP=1.0 Pa. At t=0 s, the heating by electrode is initiated. Particle flow from the inlet is separated at the Y-shaped branch. Because the thermophoresis is directed to the colder region, the PS particles cannot enter the outlet α.

Figure 3a shows the snapshot at t=0 s, at which the Joule heating of the thin-film electrode starts. According to the previous study [53] using the same electrode heater, the temperature near the electrode gradually increases with increasing time. Since the continuous phase at room temperature is supplied from the inlet, a non-uniform temperature field is formed near the electrode. The temperature field reaches an almost steady state in several seconds, producing a magnitude of the temperature gradient of about 0.6 K·μm${}^{-1}$ near the electrode. It should be noted that the fluid flow is driven by the pressure difference from the inlet to outlets α and β. As time progresses, the particle flow separation begins to be observed, as shown in Figure 3b. More specifically, it seems that microparticles cannot enter the outlet α, and the particle-concentrated region emerges near the thin-film electrode upstream. As a result, the particles are flushed out in the outlet α, and the high-concentration dispersion is obtained in the outlet β. At t=250 s, few particles exist in the outlet α, resulting in complete particle flow separation.

**Figure 4:**
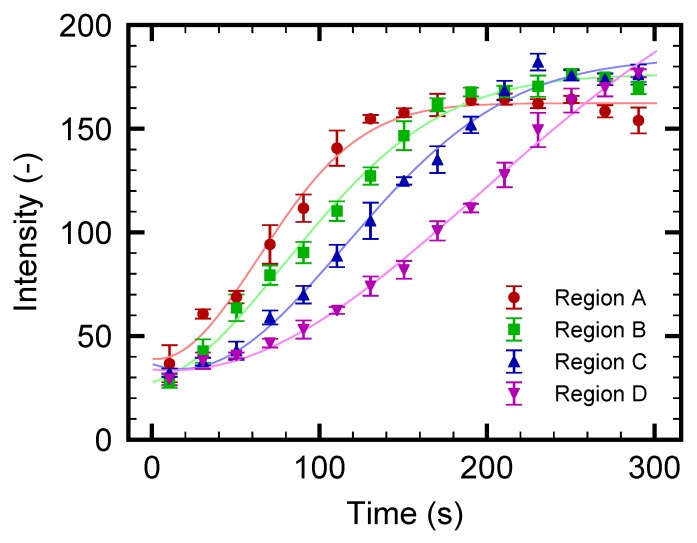
Time-development for fluorescence intensity of microparticles in the regions A, B, C, and D indicated in Figure 3a. The pressure difference ΔP is set to 1.0 Pa.

The increase in fluorescence of particular regions A, B, C, and D indicated in Figure 3a is evaluated to investigate the separation process in more detail. It should be noted that the increase in the fluorescence indicates the increase in the particle concentration. Figure 4 shows the time-development of the fluorescence intensity for these four regions. It is found that the intensity increases as time progresses for all regions; however, the onset of the increase in region A is earlier than that in region D. The time-dependent behaviors in Figure 4 are compared with the snapshots in Figure 3. At the early stage of the experiment, as shown in Figure 3b–d and Figure 4 for t≤100 s, the particles begin to accumulate in the region where the flow drag is counterbalanced by the thermophoretic force, as in [53]. At the later stage of the experiment, as shown in Figure 3d–g and Figure 4 for t≥100 s, these concentrated particles are gradually transported to the outlet β as time progresses. Therefore, the increase in the fluorescence intensity in regions B, C, and D occurs later, as shown in Figure 4. The saturation of the intensity in region A and the intensity increase in region D are caused by the transport of these particles to the outlet β.

For the concentration range investigated in the present study, no apparent particle concentration effect is observed since the initial concentration of $O(10^{-2})$ wt% is sufficiently diluted to neglect the finite-size effect and the inter-particle interaction. In the case of the straight channel of the previous study [53], the concentration increased by 100-fold to 4 wt% after 5 min of operation with a similar flow rate. When the device is operated for a longer duration, such a large concentration ratio may cause the finite-size effect and/or the inter-particle interaction that complicate the phenomena. On the contrary, the saturated particle density observed in Figure 4 is more favorable than that in the straight channel since the present device can avoid reaching too high a particle concentration even with a longer operation time. That is to say, the branched channel is suitable for practical applications where continuous particle flow separation may be required. With the aim of achieving more effective particle separation with a larger flow rate, the design of both the flow and temperature fields are important because the position of the highly concentrated particle region and the value of the saturated particle density are determined as the result of the interaction between these two fields. The present demonstration is a first step toward designing better channel and electrode patterns.

### 3.3. Nanoparticle Flow Separation

In this section, the results for nanoparticles with d=100 nm are presented. In the previous study [54], it was demonstrated that the PS nanoparticles were repelled from the hot spot produced by laser irradiation. Although the sample solution in [54] contained an additional surfactant, it was expected that the nanoparticles in the present experimental condition were also repelled from the hot region since the addition of a surfactant did not result in a reversal of the thermophoresis direction of PS microparticles in [53].

**Figure 5:**
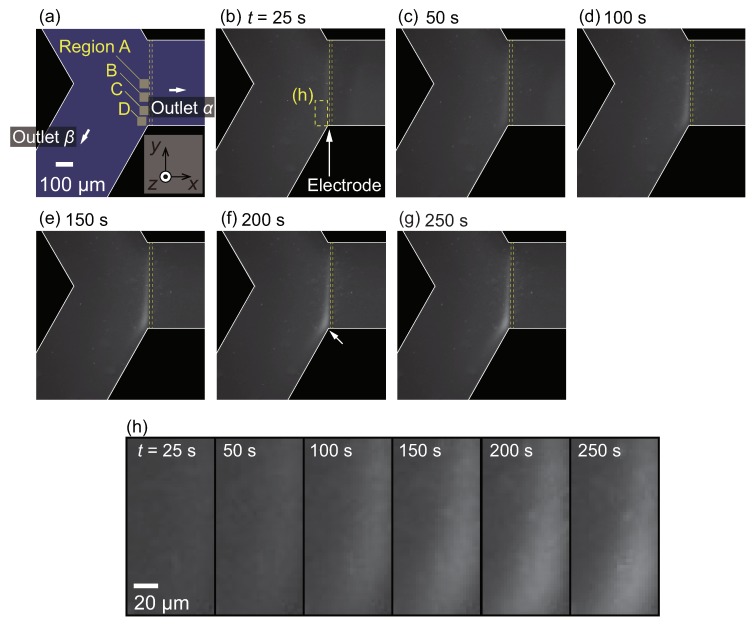
(**a**) Schematic figure of the test section and the positions of region A, B, C, and D analyzed in Figure 6; (**b**–**g**) Time series of the nanoparticle fluorescence. The particle flow separation is induced by microscale thermophoresis for the case with a particle diameter d=100 nm and ΔP=1.0 Pa. At t=0 s, the heating by the electrode is initiated. Particle flow from the inlet is separated at the Y-shaped branch. Because the thermophoresis is directed to the colder region, the PS particles cannot enter the outlet α. (**h**) Magnified figures of (**b**–**g**) for a rectangular region indicated in (**b**).

**Figure 6:**
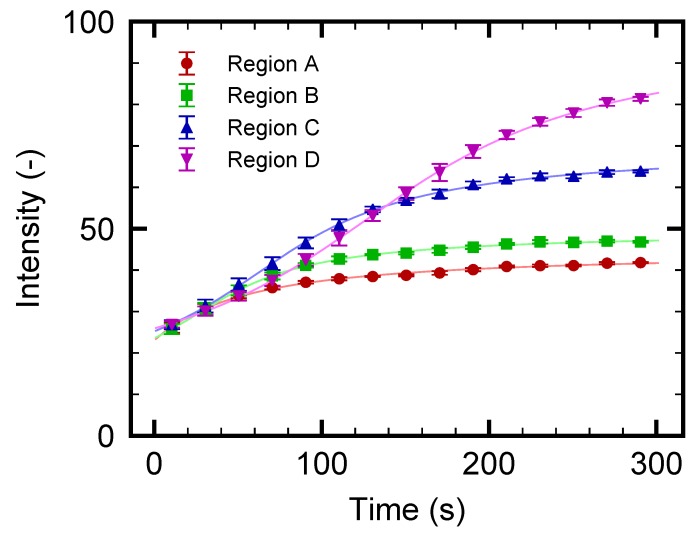
Time-development for fluorescence intensity of nanoparticles in the regions A, B, C, and D indicated in Figure 5a. The pressure difference ΔP is set to 1.0 Pa.

First, ΔP=1.0 Pa is used as in Section 3.2. Figure 5 shows the snapshots of the obtained video images. It should be noted that the nanoparticles are too small to distinguish each of them with the present optical setup. This is the reason for using florescent nanoparticles. As time goes on, the left side of the electrode starts to become bright, indicating the increase of fluorescent nanoparticles. At t≥200 s, the lower-left region near the electrode, indicated by an arrow in Figure 5f, shows an apparent increase in the fluorescence intensity. This is clearly seen from Figure 5h, which is the magnification of a rectangular region indicated in Figure 5b. The increased nanoparticle concentration is then transported to the outlet β. This behavior of nanoparticles is qualitatively similar to that of microparticles presented in Figure 3; however, the position of the particle-concentrated region is closer to the electrode in this case. Such a difference is attributed to the difference in thermophoretic mobilities between micro- and nanoparticles. These results indicate that the nanoparticles are more insensitive to the temperature gradient and/or they have larger diffusion coefficients *D* than microparticles, as expected from the Stokes–Einstein relation $D=k_{B}T/\left(3\pi \eta d\right)$, where $k_{B}$ is the Boltzmann constant and *T* is the temperature. Figure 6 shows the time-development of the fluorescence intensity in regions A, B, C, and D indicated in Figure 5a. It is found that the intensity for all the regions increases with increasing time; however, the increment is smaller than that for the experiment using microparticles, as shown in Figure 4. More specifically, the intensity is, at most, three times higher than that at the initial state t=0 s, indicating an almost three-fold increase in nanoparticle concentration. The increase of the intensity in region D, which is placed at the entrance to the outlet β, is slightly delayed but larger compared with that of other regions, because the concentrated nanoparticles in regions A, B, and C are transported to the outlet β as time progresses. The dynamic behavior of nanoparticles will be discussed in Section 3.4 using a simple model.

Comparing the results from microparticles, as shown in Figure 3 and Figure 4, with those from nanoparticles, as shown in Figure 5 and Figure 6, it can be concluded that the nanoparticles are more difficult to separate. This is because the thermal fluctuations become comparable to or stronger than the thermophoretic force and flow drag. If one wants to separate the nanoparticles more effectively, there are some methods of improvement. It should be noted that producing a larger temperature increase is not an appropriate option since it will easily cause the solution to boil if an aqueous solution is used. Firstly, downsizing the electrode width to produce a steeper temperature gradient will be effective. For this approach, the fabrication process of the electrode pattern should be improved to achieve a better yield ratio. Another approach is to use a different choice of electrolyte. It was reported in [34] that the Debye length, which is determined by the concentration of the electrolyte, affects the strength of the thermophoresis. Since the theory of thermophoresis has not been fully developed yet, such an approach will depend on trial and error. The present study can be used as a reference toward achieving more efficient nanoparticle separation using thermophoresis.

**Figure 7:**
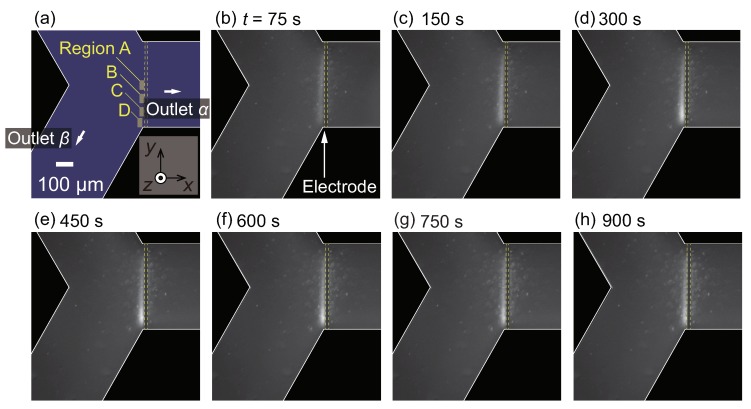
(**a**) Schematic figure of the test section and the positions of region A, B, C, and D analyzed in Figure 8; (**b**–**h**) Time series of the nanoparticle fluorescence. The particle flow separation is induced by microscale thermophoresis for the case with a particle diameter d=100 nm and ΔP=0.5 Pa. At t=0 s, the heating by electrode is initiated. Particle flow from the inlet is separated at the Y-shaped branch. Because the thermophoresis is directed to the colder region, the PS particles hardly enter the outlet α.

**Figure 8:**
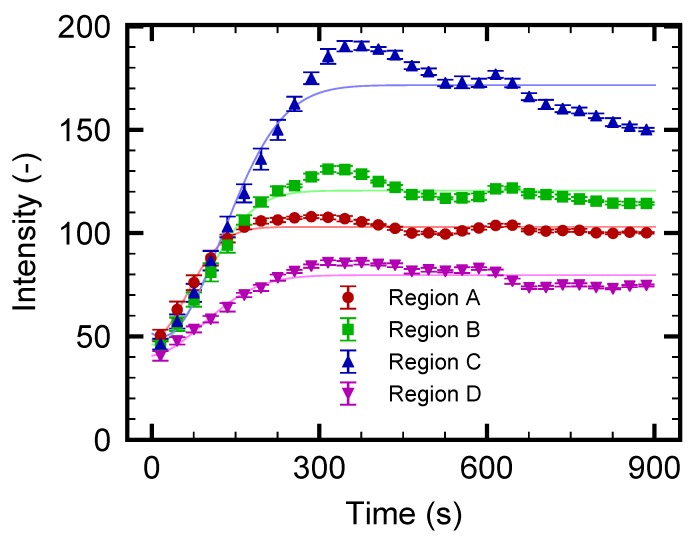
Time-development for fluorescence intensity of nanoparticles in regions A, B, C, and D indicated in Figure 7a. The pressure difference ΔP is set to 0.5 Pa.

Next, a similar experiment for the nanoparticles using a smaller pressure difference ΔP=0.5 Pa is carried out with a longer experimental duration of 900 s. The intention here is to increase the nanoparticle concentration near the electrode using a slower mean flow of the continuous phase. It should be noted that the slower flow speed is expected to prevent the transport of the concentrated particles into the outlet β. The result is presented in Figure 7. The fluorescence in the left side of the electrode starts to increase as in the previous case with ΔP=1.0 Pa, as shown in Figure 5. At a first glance, the increase of the fluorescence intensity is enhanced due to the slower flow speed. However, it seems that the intensity increase converges to a constant value at a later stage of the experiment, as shown in Figure 7f–h. Then, the time-development of the fluorescence intensity in regions A, B, C, and D as indicated in Figure 7a is investigated. Figure 8 presents the intensity for these four regions. First, it is found that the intensity for region C is the most prominent. This trend is different from that in Figure 6, where the intensity of region D is the largest. This is attributed to the slower flow velocity component in the *y* direction, which transports the particles from region A to the negative *y* direction. The intensity for regions A, B, and D becomes saturated and fluctuated slightly. The fluctuation is caused by the occasional leak of trapped nanoparticles into the outlet α. Such a leak may be attributed to the longer experimental duration, which may cause an increase in the overall temperature in the entire microfluidic channel. Recall that the thermophoresis is driven by the temperature gradient, which may be diminished by the increase of the entire temperature. The result from region C, at which the increase of the intensity is the most prominent, shows that the intensity starts to decrease after t=300 s. These results indicate that the effect of thermophoresis diminishes as time goes on, which also supports the speculation stated above regarding the entire temperature increase. To avoid the entire increase in the temperature, the use of different material for the channel wall is proposed. For instance, a Si substrate instead of the present glass substrate will dissipate the heat more efficiently, resulting in a more stable temperature field. A better design of the device which reduces such a leak will be explored in the future studies. Despite the remaining challenges, it is concluded that the concept of nanoparticle flow separation is demonstrated successfully using the present microfluidic device.

### 3.4. Numerical Modeling for Nanoparticle Distribution

**Figure 9:**
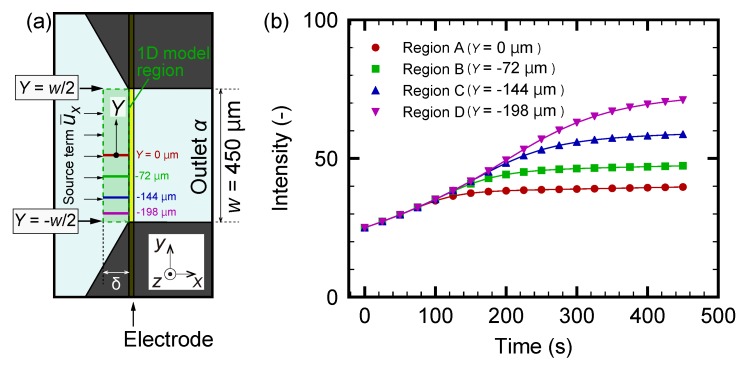
(**a**) Schematic of the numerical model on the concentration increase at the branch. (**b**) Numerical results regarding the time-development for fluorescence intensity of nanoparticles in the regions A (Y≈0 μm), B (Y≈−72 μm), C (Y≈−144 μm), and D (Y≈−198 μm), shown in panel (**a**) and corresponding to Figure 6, where Y=0 μm is placed at the center of the outlet α in the *Y* direction.

In this section, a simple model is proposed to explain the dynamics of the nanoparticle distribution observed in the experiment detailed in Figure 5 and Figure 6. A rectangular region near the left side of the electrode is focused, as shown in Figure 9a. The width δ is smaller than *w*, and we consider the number density of the nanoparticles is uniform in the *x* direction. More precisely, a one-dimensional convection diffusion equation along the *Y* axis, that is, the left side of the electrode heater, is considered. It should be noted that *Y* is used for the 1D model in Figure 9a, instead of *y* in the original coordinates. Let N(Y,t) be the number density of the nanoparticles with a time variable *t*. Velocity vectors of the continuous phase are denoted by $\left(u_{x},u_{y}\right)$ in the xy plane. These quantities are obtained from the experimental results and thus are given functions in the following numerical model. Due to the temperature gradient produced by the heater, thermophoresis of the particles with the velocity $u_{T}=-D_{T}\left(\frac{\partial T}{\partial x}\right)$ is induced in the *x* direction, where $D_{T}$ is the thermophoretic mobility. As described in the previous study [54], $D_{T}$ was estimated to be almost $1\times 10^{-12}$ m${}^{2}$· s${}^{-1}$·K${}^{-1}$, that is, the thermophoretic velocity was directed toward the colder region. The temperature gradient $\frac{\partial T}{\partial x}$ was estimated from the previous study [53] to be $\frac{\partial T}{\partial x}=0.6\times 10^{6}$ K·m${}^{-1}$. It should be noted that $u_{T}$ is considered to be uniform with respect to *Y*, and the effective speed of particle transport in the *x* direction can be written as $U_{x}\left(Y\right)=u_{x}\left(Y\right)-u_{T}$. Using these notations, the governing equation for *N* can be written as

(1) $$\frac{\partial N}{\partial t}+\frac{\partial (Nu_{y})}{\partial Y}+D\frac{\partial^{2}N}{\partial Y^{2}}=S\left(Y\right),\;S\left(Y\right)=N_{0}U_{x}\left(Y\right)/L_{0},$$

where *S* is a source term that represents the supply of nanoparticles to the computational domain, $N_{0}$ is a uniform initial value of *N*, *D* is a diffusion coefficient, and $L_{0}$ is the length of the simulation region in the *x* direction. The flow velocity $\left(u_{x},u_{y}\right)$ is obtained from the PIV data. More specifically, the values presented in Figure 2a at x=−100 μm and |y|<w/2=225 μm are used, and a least-squares fit is made to determine $\left(u_{x},u_{y}\right)$. *D* is obtained from the Stokes–Einstein relation, that is, $D=k_{B}T/\left(3\pi \eta d\right)$, where T=360 K is the temperature near the electrode heater at the steady state, $\eta =3.2\times 10^{-4}$ Pa·s [60] is the viscosity at temperature *T*, and the particle diameter is d=100 nm, as in the experiments. It should be noted that the steady temperature field is used for the modeling because an almost steady state was established after several seconds of heating [53].

Boundary conditions for Equation (1) are given as

(2) $$\begin{array}{c} \partial N/\partial Y=0,\;(Y=w/2), \end{array}$$

(3) $$\begin{array}{c} \partial N/\partial t=S\left(Y\right),\;\left(Y=w/2\right)\;\to \;N\left(Y=w/2\right)=N_{0}+\left(N_{0}U_{x}\left(Y\right)/L_{0}\right)t, \end{array}$$

where Equation (3) is introduced by assuming that $\partial u_{y}{/\partial Y|}_{Y=w/2}=0$. The boundary conditions (2) and (3) physically mean that *N* and $\left(u_{x},u_{y}\right)$ for Y>w/2, i.e., in the inlet channel, are uniform, respectively. Equation (3) results in the increase of particle density at a constant rate due to the source term.

The initial condition is uniform with the bulk value $N\left(Y,0\right)=N_{0}$. Since the number of particles is linearly correlated to the fluorescence intensity in the experiments, *N* and $N_{0}$ can be interpreted as the fluorescence intensity at *Y* and *t* and that for the initial state. For $L_{0}$, the value $L_{0}=1.0\times 10^{4}$ μm is used, which is chosen so that the model gives a result with a similar order of magnitude as the experiment. This is larger than the actual value of O(100) μm, as shown in Figure 9a. The overestimation in the model may be due to the crude approximation made for the source term *S*. Nonetheless, the model is considered to be suitable for use as the first step in the qualitative investigation of the particle distribution made below. This model can be solved using a standard finite-difference scheme.

The numerical results are shown in Figure 9b, where the time-development of the intensity, which is scaled so that the initial value is consistent with the experimental results given in Figure 6, is presented for several positions *Y*. Here, Y=0, −72, −144, and −198 μm correspond to regions A, B, C, and D in the experiments, as indicated in Figure 9a. By comparing the experiment in Figure 6 and the simulation in Figure 9b, a qualitative agreement is found; however, the range of times is different in these figures. From the simulation, the dynamic behavior of the nanoparticle concentration can be explained as follows. The nanoparticles are supplied to the left-side region of the electrode heater by the flow $u_{x}$ of the continuous phase. Due to the thermophoresis, the nanoparticles cannot go beyond the heated electrode, and are concentrated in its left-side region. This is the reason for the overall increase in the intensity that can be observed in Figure 9b. As *Y* decreases, $u_{y}$ also decreases, as shown in the PIV result of Figure 2a. Therefore, an increased particle concentration is transported in the negative *Y* direction. This leads to the larger concentration increase rate for region D compared with that for A. The present model indicates that the formation of a more concentrated region near the electrode is related to the spatial distribution of $\left(u_{x},u_{y}\right)$, and the design of the flow field at the branch is important for the control of the nanoparticle distribution.

## 4. Concluding Remarks

In the present study, a microfluidic channel with a Y-shaped branch, at which an inlet flow was separated into two symmetric outlet flows, has been developed. A thin-film electrode heater was fabricated at the entrance of one of the outlets to induce a local temperature rise for microscale thermophoresis of dispersed particles. Since the particles were repelled from the hot region, only the solvent entered the outlet with the heater, and the micro- and nanoparticles were transported to the other outlet. A simple model for the nanoparticle distribution based on the convection diffusion equation, which includes the effect of thermophoresis, was introduced, and a qualitative agreement with the experimentally observed nanoparticle motion was obtained. In this way, the particle flow separation using the microscale thermophoresis was demonstrated at the branched channel.

It resulted that the flow and temperature profiles were important to understand the detail of the particle behavior at the branch, such as the formation of a highly concentrated region. This was not shown in the previous study [53], and is an important finding of the present experiment. Therefore, the design of the device should be improved for the systematic investigation toward optimal particle flow control. Some fundamental research on flow separation, including the effect of heat transfer, was also presented in [61,62], where a backward facing step was considered instead of branched channels. In this research, the formation of recirculation zone, flow separation, and reattachment was investigated. The effect of thermophoresis on such flow geometries will be another interesting direction of thermophoretic particle flow separation. The theoretical aspects of the underlying physics of microscale thermophoresis and the above-mentioned systematic experiments will be carried out in future studies.
